# Butylphthalide Inhibits Autophagy and Promotes Multiterritory Perforator Flap Survival

**DOI:** 10.3389/fphar.2020.612932

**Published:** 2021-01-29

**Authors:** Baolong Li, Zhengtai Chen, Xiaobin Luo, Chenxi Zhang, Hongyu Chen, Shuxuan Wang, Mengyao Zhao, Haiwei Ma, Junling Liu, Mengshi Cheng, Yanyan Yang, Hede Yan

**Affiliations:** ^1^ Department of Orthopaedics, The Second Affiliated Hospital and Yuying Children’s Hospital of Wenzhou Medical University, Wenzhou, China; ^2^ Key Laboratory of Orthopaedics of Zhejiang Province, Wenzhou, China; ^3^ The Second School of Medicine, Wenzhou Medical University, Wenzhou, China; ^4^ Key Laboratory of Pediatric Hematology and Oncology Ministry of Health, Pediatric Translational Medicine Institute, Shanghai Children’s Medical Center, Shanghai Jiao Tong University School of Medicine, Shanghai, China; ^5^ Respiratory Medicine, The First Affiliated Hospital of Wenzhou Medical University, Wenzhou, China; ^6^ Infectious Medicine, The First Affiliated Hospital of Wenzhou Medical University, Wenzhou, China; ^7^ The First Clinical Medical College of Wenzhou Medical University, Wenzhou, China

**Keywords:** multiterritory perforator flap, butylphthalide, Autophagy, Angiogenesis, apoptosis

## Abstract

Multiterritory perforator flap is an important plastic surgery technique, yet its efficacy can be limited by partial necrosis at the choke Ⅱ zone. Butylphthalide (NBP) has been used for many diseases but has not been studied in the multiterritory perforator flap. With the effect of NBP, we observed increasing in capillary density, inhibition of autophagy and oxidative stress, and a reduction in apoptosis of cells, all consistent with increased flap survival. However, the protective effect of NBP on multiterritory perforator flap was lost following administration of the autophagy agonist rapamycin (Rap). Through the above results, we assumed that NBP promotes flap survival by inhibiting autophagy. Thus, this study has found a new pharmacological effect of NBP on the multiterritory perforator by inhibiting autophagy to prevent distal postoperative necrosis and exert effects on angiogenesis, oxidative stress, and apoptosis within the flap.

## Introduction

Soft tissue reconstruction is a central component of plastic surgery (Luo et al., 2020). The multiterritory perforator is a large area flap with independent arterial and venous perforator nourishment (Lin et al., 2020). Flap design versatility can reduce the number of donor sites needed and best resembles anatomical aspects of a microvascular flap, leading the multiterritory perforator flap to replace the traditional flap in clinical practice (Fichter et al., 2016; Guo et al., 2018). However, necrosis of the distal flap is a common postoperative complication (Chen et al., 2018) as angiogenesis is insufficient to support the growth and metabolism of distal tissues, leading to tissue level ischemic necrosis (Chen et al., 2017). This can occur after the perforating branch is established and blood vessels begin to grow distally through pedicle blood vessels (Wang et al., 2017). Simultaneously, recanalization of blood in the flap can lead to ischemia-reperfusion injury and further exacerbates edema and necrosis of the distal end of the flap (Chen et al., 2018). Apoptosis caused by reperfusion or long-term ischemia is also thought to contribute to complications following a multiterritory perforator flap procedure (Li et al., 2019). Prior studies have shown that inhibition of oxidative stress and apoptosis can reduce the risk of developing ischemia-reperfusion injury and distal necrosis of the flap (Lin et al., 2018).

Autophagy is considered a self-protective mechanism in higher organisms (Hou et al., 2020). In the process of autophagy, lysosomes selectively degrade and remove damaged and senescent cells, along with excess biological macromolecules and organelles, before finally releasing free small molecules for collective recycling (Yu et al., 2014). Recently, the effect of inhibiting autophagy on the body has received widespread attention. Inhibition of autophagy has been shown to prolong the survival time of allogeneic liver transplant patients by promoting the apoptosis of pathogenic CD8^+^ T cells (Chen et al., 2019). Further beneficial effects of autophagy inhibition come from studies showing that inhibiting autophagy enhances viability of perforator flaps and promotes angiogenesis (Jin et al., 2018). On the basis of these observations, we sought to identify an agent that could promote angiogenesis, oxidative stress, and apoptosis and inhibit autophagy to improve the survival of multiterritory perforator flap.

This aim led us to study DL-3-n-butyl phthalate (NBP), a compound derived from celery seeds that is commonly used in the treatment of stroke patients (Ye et al., 2019). NBP promotes angiogenesis in ischemic diseases by activating the ERK1/2 and phosphatidylinositol 3-kinase (PI3K)/AKT/eNOS signaling pathways (Ye et al., 2019). NBP has also been shown to inhibit apoptosis through the MAPK pathway and protect the brain of Aβ1-42-treated rats (Song et al., 2017). Zhu et al. have also reported that NBP can inhibit oxidative stress and delays the occurrence and development of diabetic cataracts (Zhu et al., 2018). NBP is also known to inhibit autophagy and apoptosis by activating AKT/mTOR signaling and regulating protein expression. It has also been shown to reduce neurologic deficits in a mouse model of repeated cerebral ischemia and reperfusion (Zhong et al., 2019). However, NBP effects in multiterritory perforator flap are unknown.

In this study, we explore the effects of NBP on autophagy within perforator flaps and study autophagy-mediated effects on angiogenesis, oxidative stress, and apoptosis within a multiterritory perforator flap.

## Results

### NBP Improves Survival of Multiterritory Perforator Flap

**FIGURE 1:**
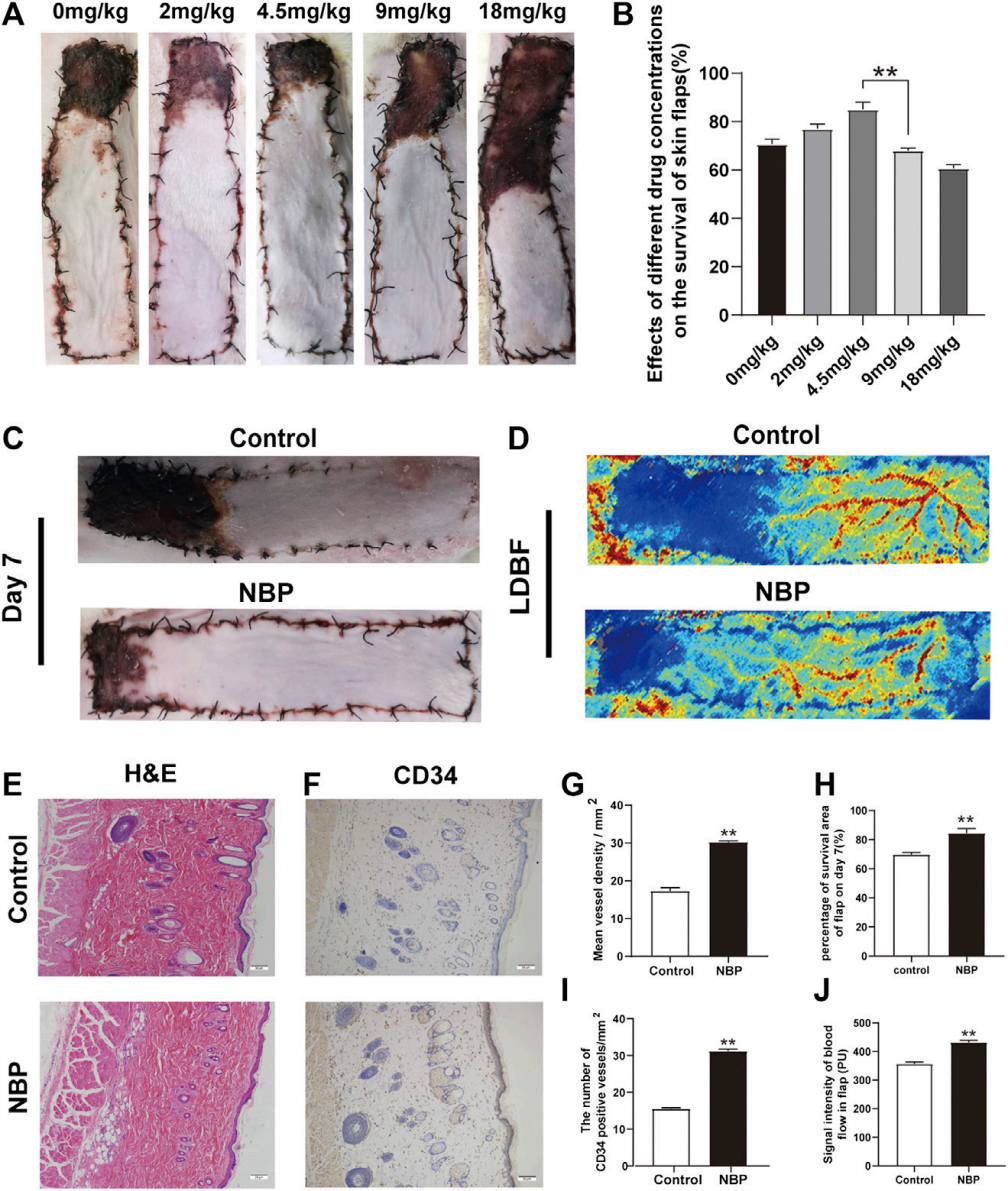
NBP improves survival of multiterritory perforator flap. **(A)** Determine the best dose of the drug by the survival area of rat skin flap. **(B)** Digital images of control and NBP-treated rats on POD 7. **(C)** Percentage of survival area on POD 7. **(D)** LDBF in each group on POD 7. **(E)** Percentage of blood flow signal intensity within the flap. **(F)** H&E staining of blood vessel density in the control and NBP groups (200X); scale bar, 50 μm. **(G)** IHC staining for CD34 in the choke Ⅱ zone of the control and NBP groups (200X); scale bar, 50 μm. **(H)** Percentage of microvascular density (MVD) in each group. **(I)** Percentage of CD34 positive vessels. Values are shown as mean ± SEM, *n* = 6 per group. *p < 0.05 and **p < 0.01 vs. control group.

Under different concentrations of NBP treatment, it can be found from the statistical graph that the survival area of the flap is increasing at 2 and 4.5 mg/kg. After 4.5 mg/kg, as the concentration increased, the survival area of the flap decreased. 4.5 mg/kg is the best dose for flap survival. Therefore, it was used as the experimental dose in subsequent experiments. (Figures 1A,B). Seven days after the operation, the choke Ⅱ zone and pedicle of the flap showed signs of darkening, hardening, and necrosis. NBP-treated animals had a superior mean survival area (control: 71.19 ± 1.30%; NBP: 84.28 ± 1.85%; control vs. NBP: *p* = 0.002) and improved LDBF compared with control-treated animals (Figures 1C,H,J). H&E results showed that the NBP-treated group had a significant increase in the number of microvessels, and the average vascular density was higher compared with the control group (Figures 1E,G), suggesting improved angiogenesis. Furthermore, NBP-treated animals also had more CD34^+^ cells in their blood vessels compared with controls (Figures 1F,I). Collectively, these data suggest that NBP can improve the survival of multiterritory perforator flap.

### NBP Improves Angiogenesis in Multiterritory Perforator Flap

**FIGURE 2:**
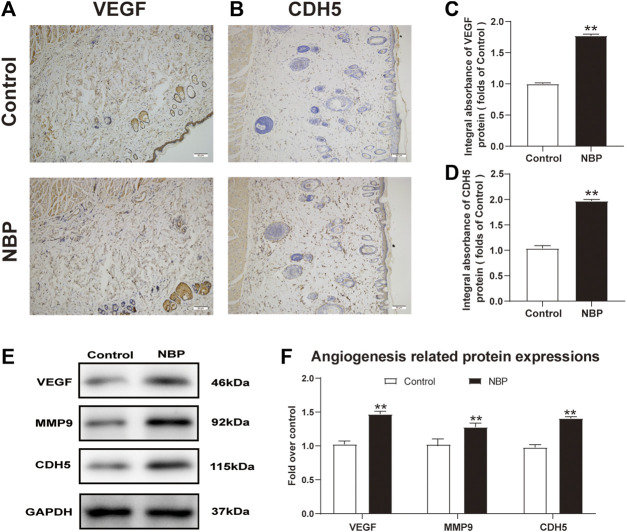
NBP improves angiogenesis in multiterritory perforator flap. **(A)** and **(B)** IHC of VEGF and CDH5 in the ischemic flap of the control and NBP-treated rats. **(C)** and **(D)** Optical density values of VEGF and CDH5. **(E)** Western blotting of MMP9, VEGF, and CDH5 in control and NBP-treated groups. **(F)** Optical density values of MMP9, VEGF, and CDH5 from western blot. Gels were run under similar experimental conditions and cropped edited only for clarity. Values are shown as mean ± SEM, *n* = 6 per group. *p < 0.05 and **p < 0.01 vs. control group.

Angiogenesis is critical for the survival of a multiterritory perforator flap. We evaluated expression of the angiogenic factors such as vascular endothelial growth factor (VEGF) and Cadherin5 (CDH5) using immunohistochemistry (IHC) results and found that NBP-treated rats had more VEGF and CDH5 positive cells than controls (Figures 2A–D). Western blot analysis also confirmed elevated protein expression of these two factors in NBP-treated animals as well as MMP9 (Figures 2E,F) and further demonstrated that NBP is associated with improved angiogenesis in multiterritory perforator flap.

### NBP Attenuates Apoptosis in Multiterritory Perforator Flap

**FIGURE 3:**
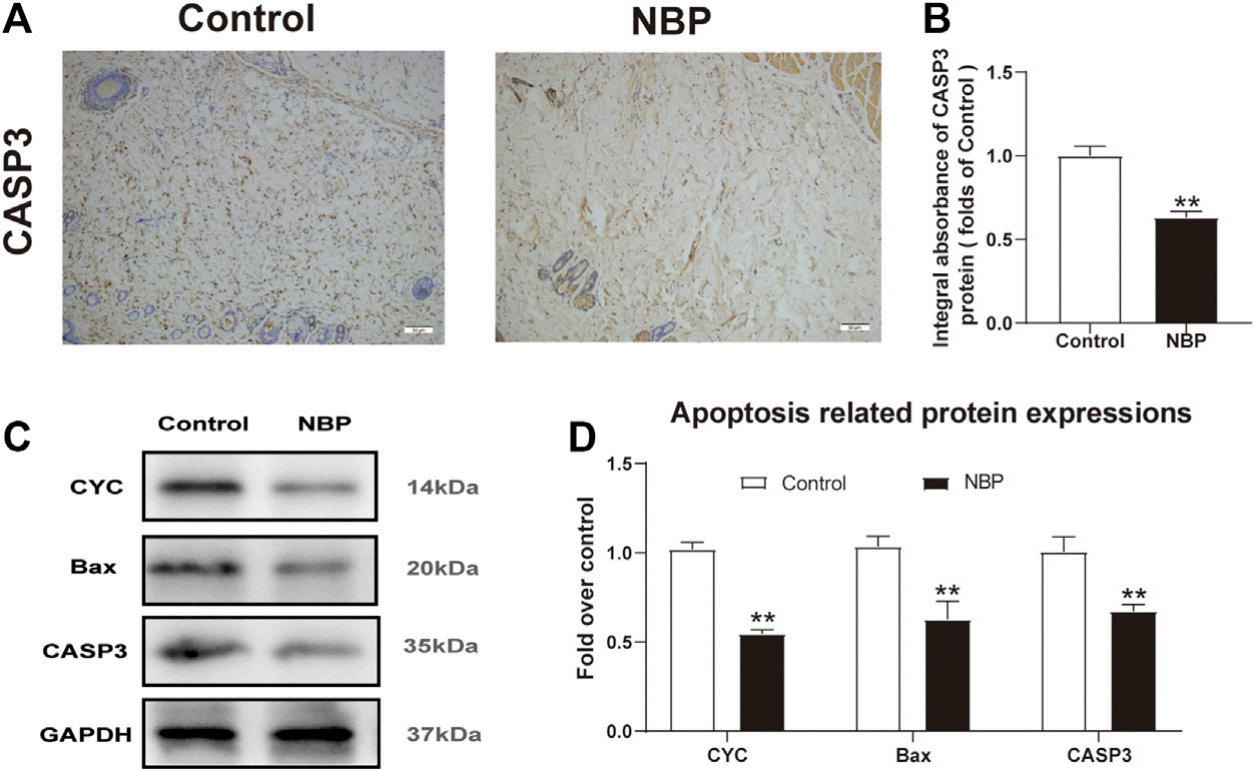
NBP attenuates apoptosis in multiterritory perforator flap. **(A)** IHC for CASP3 in the control and NBP groups (200X); scale bar, 50 μm. **(B)** CASP3 optical density for each group. **(C)** Western blotting of CYC, Bax, and CASP3. **(D)** Optical density of CYC, Bax, and CASP3 in each group. Gels were run under similar experimental conditions and cropped edited only for clarity. Values are shown as mean ± SEM, *n* = 6 per group. *p < 0.05 and **p < 0.01 vs. control group.

Next, we investigated NBP effects on apoptosis in multiterritory perforator flap. Using IHC, we found significantly reduced CASP3 staining in the dermis of the NBP-treated rats compared with controls (Figures 3A,B). Similarly, western blotting for Bax and CYC showed a significant reduction in protein expression of NBP-treated animals vs. controls (Figures 3C,D) which, along with the IHC results, suggests NBP reduces apoptosis in multiterritory perforator flap.

### NBP Attenuates Oxidative Stress in Multiterritory Perforator Flap

**FIGURE 4:**
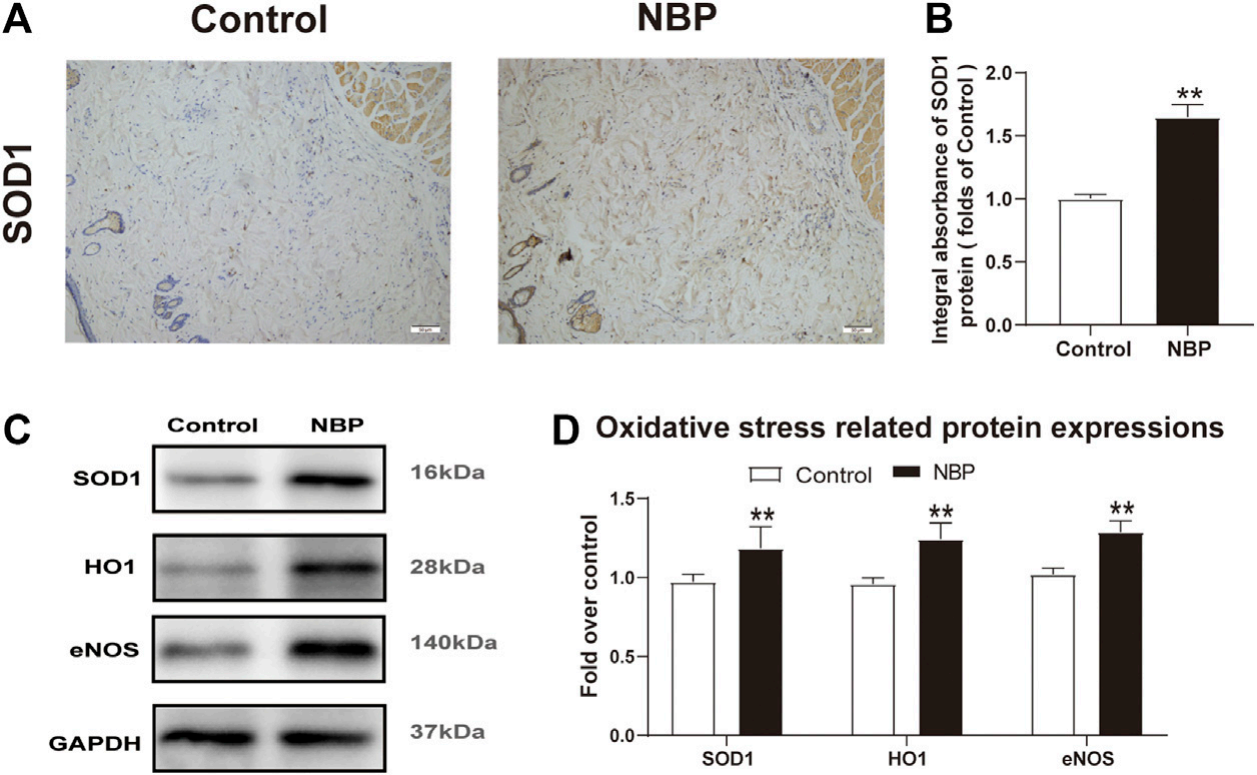
NBP attenuates oxidative stress in multiterritory perforator flap. **(A)** IHC of SOD1 in each group (200X); scale bar, 50 μm.**(B)** Optical density values of SOD1 in each group. **(C)** Western blotting for SOD1, HO1, and eNOS in each group. **(D)** Optical density values of SOD1, HO1, and eNOS in each group. Gels were run under similar experimental conditions and cropped edited only for clarity. Values are shown as mean ± SEM, *n* = 6 per group. *p < 0.05 and **p < 0.01 vs. control group.

Oxidative stress is known to have a significant effect on flap survival. We evaluated NBP effects on oxidative stress through analysis of SOD1, which is a sign of the oxidative stress in area II of the flaps. Both IHC and western blot showed that SOD1 levels were significantly elevated in the NBP group compared with controls (Figures 4A–D). Likewise, HO-1 and eNOS levels were also improved in the NBP-treated animals (Figures 4C,D). These findings suggest that NBP may improve skin flap survival by reducing oxidative stress.

### NBP Inhibits Autophagy in Multiterritory Perforator Flap

**FIGURE 5:**
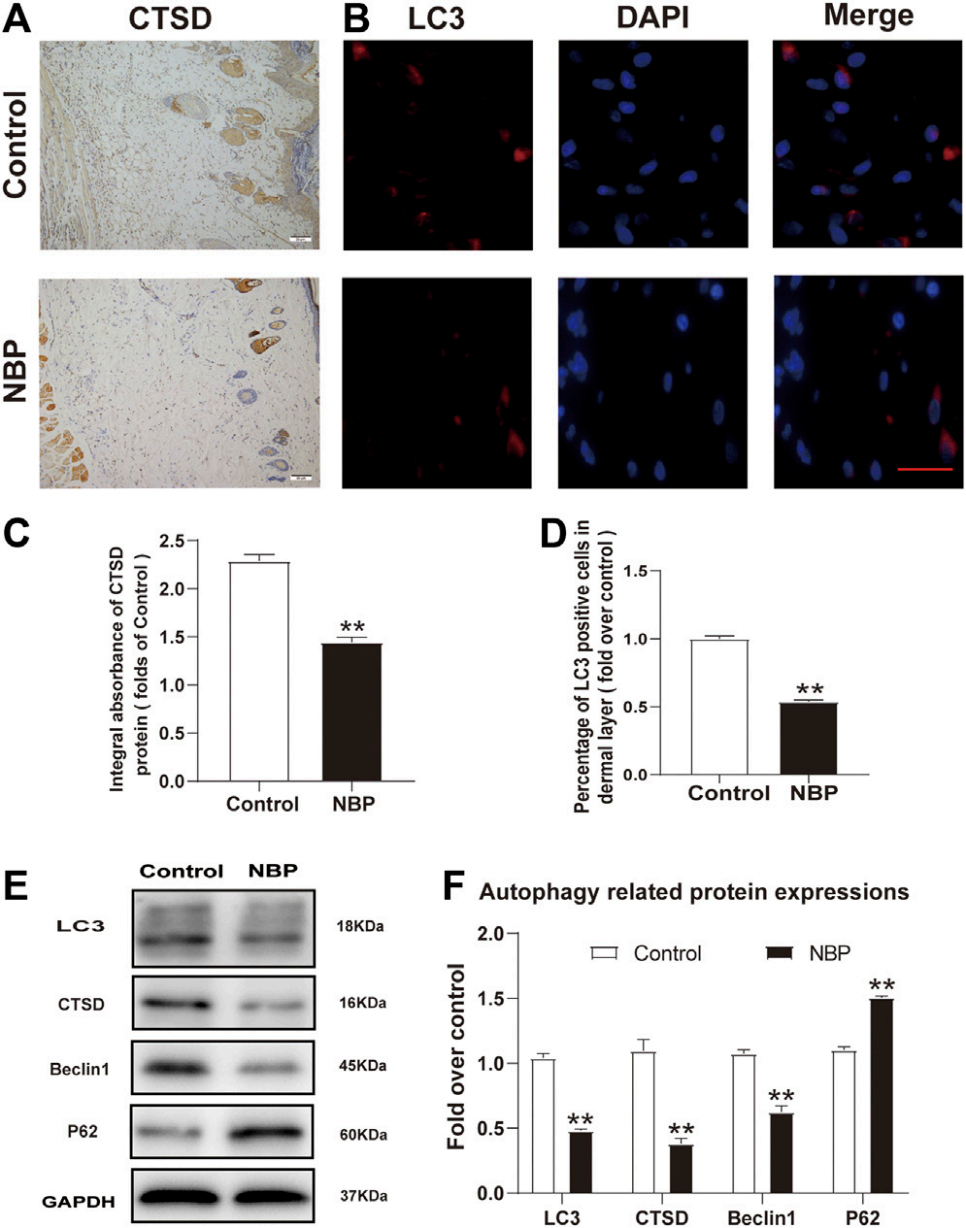
NBP inhibits autophagy in multiterritory perforator flap. **(A)** IHC for CTSD in the ischemic flap of the control and NBP-treated animals (200X); scale bar, 50 μm. **(B)** Autophagosome (LC3, red) immunofluorescent staining of cells in the choke Ⅱ zone in the control and NBP groups. Nuclei are counterstained with DAPI (blue) (scale bar, 20 μm). **(C)** Optical density values of CTSD in each group. **(D)** Percentage of LC3-positive cells in each group. **(E)** and **(F)** Western blot for Beclin1, CTSD, SQSTM1/p62, and LC3 in the ischemic flap of the control and NBP groups. Gels were run under similar experimental conditions and edited only for clarity. Values are shown as mean ± SEM, *n* = 6 per group. *p < 0.05 and **p < 0.01 vs. control group.

We also examined NBP effects on autophagy by examining the protein expression levels of LC3, Beclin1, CTSD, and p62. Immunofluorescence staining showed a decrease in LC3 positive cells in NBP-treated rats compared with controls (Figures 5B,D). Conversely, IHC and western blot analysis of CTSD showed reduced levels in the NBP-treated group (Figures 5A,C,E,F). Protein levels of Beclin1 and LC3 were also decreased in the NBP group (Figures 5E,F). However, p62 protein levels increased following NBP treatment (Figures 5E,F). Thus, NBP appears to promote flap survival by inhibiting autophagy.

### Rap Reverses Effects of NBP on Angiogenesis, Oxidative Stress, and Apoptosis in Multiterritory Perforator Flap

**FIGURE 6:**
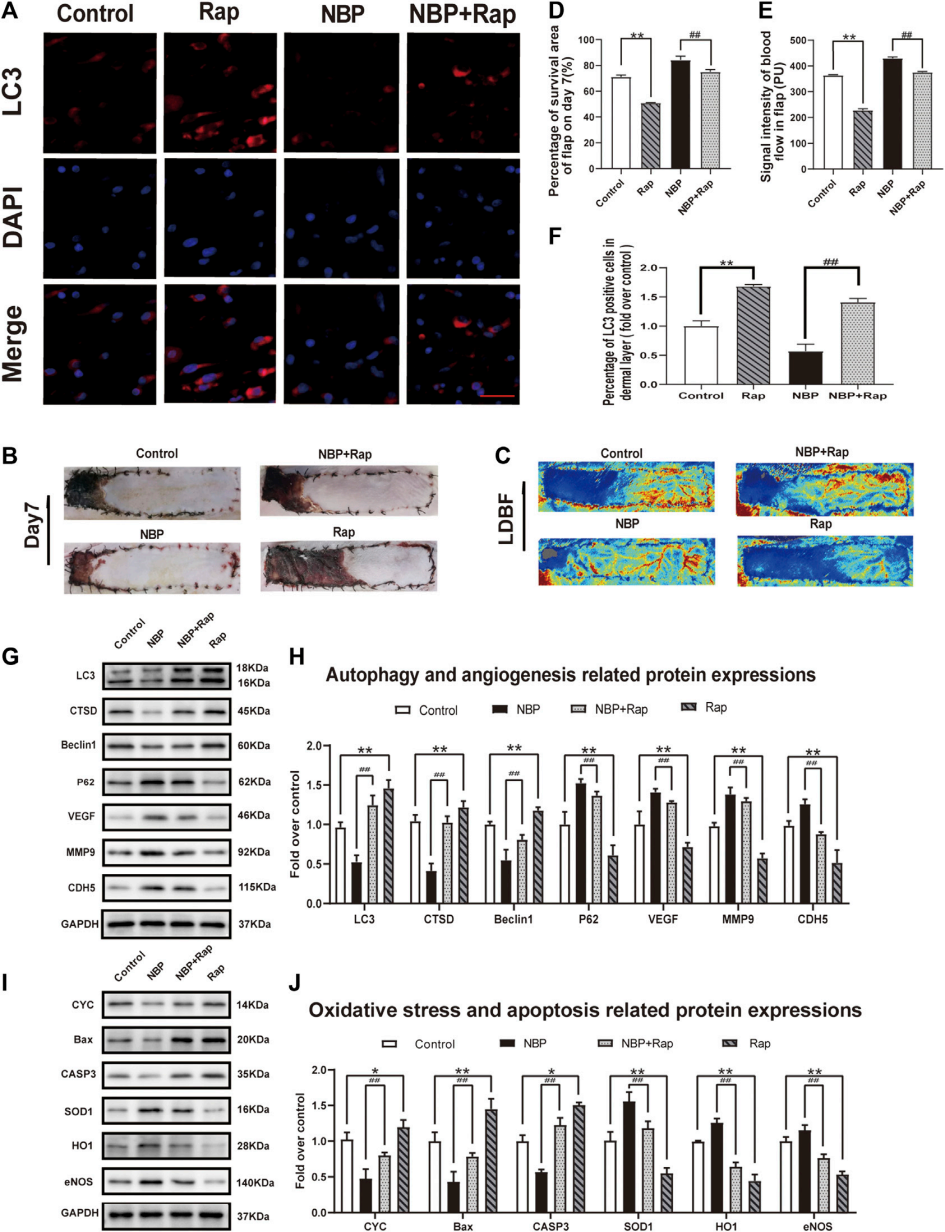
Rapamycin reverses effects of NBP on angiogenesis, oxidative stress, and apoptosis in multiterritory perforator flap. **(A)** Autophagosomes (LC3, red) in cells in the control, NBP, NBP + rapamycin, and rapamycin groups. Nuclei are counterstained with DAPI (blue) (scale bar, 20 μm). **(B)** Digital images of the control, NBP, NBP + rapamycin, and rapamycin groups on POD 7. **(C)** LDBF in each group on POD 7. **(D)** Percentage of survival area on POD 7. **(E)** Percentage of the signal intensity of blood flow within the flap in each group. **(F)** LC3 positive cells in each group. **(G)** Autophagy-related protein expression (LC3, CTSD, Beclin1, and SQSTM1/p62) and angiogenesis-related proteins (VEGF, MMP9, and CDH5). **(H)** Optical density of LC3, CTSD, Beclin1, SQSTM1/p62, VEGF, MMP9, and CDH5 in each group. **(L)** Apoptosis-related protein expression (CYC, Bax, and CASP3) and oxidative stress-related protein expression (SOD1, HO1, and eNOS) in each group. **(M)** Optical density of CYC, Bax, CASP3, SOD1, HO1, and eNOS expressions in each group. Gels were run under similar experimental conditions and edited only for clarity. Values are shown as mean ± SEM, *n* = 6 per group. *p < 0.05 and **p < 0.01 vs. control group; #*p* < 0.05 and ##*p* < 0.01 vs. NBP group.

We sought to untangle the wide-ranging effects of NBP on angiogenesis, oxidative stress, apoptosis, and autophagy by testing the effect of NBP administration with rapamycin, a known autophagy inducer. In rats treated with NBP and rapamycin, we observed a significant increase in LC3 positive cells vs. NBP alone (Figures 6A,F). The frequency of LC3 positive cells was also increased in animals treated with rapamycin alone compared with controls (Figures 6A,F). Western blotting showed that compared with NBP monotherapy, NBP + Rap reduced p62 expression and increased Beclin1, CTSD, and LC3 expression (Figures 6G,H). Rapamycin monotherapy had a similar effect on these autophagy markers (Figures 6G,H). These data suggest that rapamycin-mediation autophagy activation reverses NBP-mediated autophagy inhibition. Our angiogenesis analysis showed a significant down-regulation of MMP9, VEGF, and CDH5 in the NBP + Rap group compared with NBP alone and in the NBP group compared with controls (Figures 6G,H). We observed similar effects on oxidative stress, evidenced by decreased protein expression levels of eNOS, SOD1, and HO1 in NBP + Rap-treated rats compared with NBP alone and in single agent NBP-treated animals compared with controls (Figures 6L,M). Conversely, CYC, Bax, and CASP3 expression were significantly increased in the NBP + Rap group vs. NBP alone and in the Rap group compared with controls (Figures 6L,M), suggesting increased apoptosis.

Overall effects on flap survival were similar as the Rap-treated group and showed lower survival compared with controls, and NBP + Rap-treated animals had lower survival vs. NBP alone (control: 71.19 ± 1.30%; Rap: 50.95 ± 1.25%; NBP: 84.28 ± 1.85%; NBP + Rap:75.02 ± 1.87%; respectively; control vs. Rap: *p* ≤ 0.002; NBP vs NBP + Rap: *p* ≤ 0.004 Figures 6B,D). LDBF was also decreased in rapamycin-treated rats vs. controls, and the NBP + Rap group had less blood flow than the NBP alone group (Figures 6C,E). Taken together, our data suggest that the mechanism of NBP improvement of skin flap survival is via autophagy inhibitor and effects on oxidative stress, apoptosis, and angiogenesis.

## Discussion

Coverage of skin and soft tissue defects with different flap is a common procedure in the field of reconstructive plastic surgery (Fujioka, 2014; Fichter et al., 2016; Zhang Y et al., 2016). Prior studies have examined various interventions aimed at improving flap survival (Zhou et al., 2019). Despite these efforts, partial or total flap ischemic necrosis continues to be a prevalent clinical problem. NBP is a compound extracted from celery seeds and is widely used for the treatment of ischemic stroke (Ye et al., 2019) and has shown the ability to promote angiogenesis, inhibit apoptosis, attenuate oxidative stress, and down regulate autophagy (Xiong et al., 2017; Zhong et al., 2019). To our knowledge, this is the first study examining the effects of NBP in the context of a multiterritory perforator flap.

One goal of interventions promoting flap survival is the improvement of angiogenesis. Angiogenesis is a complex process in which MMP9 degrades proteins related to vascular wall stability to disrupt preexisting cell connections (Mărginean et al., 2019). VEGF then promotes endothelial cell migration, proliferation, and angiogenesis via the PI3K/AKT and RAF/MEK-ERK pathway (Cai et al., 2015). Additionally, vascular endothelial CDH5 is specifically expressed in endothelial cell adhesion junctions and plays an important role in intercellular adhesion and signal transduction (Yang et al., 2018). We found that NBP significantly increased the number of microvessels in multiterritory perforator flap which was corroborated by increased CD34 cell staining by H&E, improved LDBF values, and increased expression of VEGF, CDH5, and MMP9. Therefore, we speculate that NBP promotes neovascularization in the dermis of the multiterritory perforator flap.

Mechanisms of tissue damage in multiterritory perforator flap related to ischemia-reperfusion involve induction of oxidative stress and cell apoptosis (Wang Z et al., 2018) whereby reactive oxygen species (ROS) destroy cell membranes, nucleic acids, and chromosomes (Gupta et al., 2019). Cellular death is further exacerbated by ROS-mediated disruption of cell membranes through lipid peroxidation (Zhao et al., 2016). Antioxidants like SOD1, HO1, and eNOS aid in relieving oxidative stress (Arise et al., 2012; Li et al., 2018). NBP has also been shown to reduce oxidative stress (Liu et al., 2017). Increased expression of SOD1, eNOS, and HO1 in the dermis of the NBP-treated animals compared with controls suggests NBP may have alleviated oxidative stress caused by ischemia-reperfusion in the setting of multiterritory perforator flap.

Programmed cell death occurs when Bax induces mitochondrial outer membrane permeability and swelling, CYC is released from mitochondria to form apoptotic bodies, and caspase three induces apoptosis (Shi et al., 2015). Apoptosis also occurs in the skin flap after ischemia-reperfusion injury (Marunouchi and Tanonaka, 2015). Our study found that NBP significantly inhibited the expression of Bax, CYC, and CASP3, suggesting NBP can promote multiterritory perforator flap survival by reducing apoptosis.

Autophagy is the primary intracellular degradation system (Hou et al., 2020). Beclin1 and LC3 are used as representative molecules indicating autophagosome formation (Fimia et al., 2007; Mizushima et al., 2011), whereas CTSD is characteristic of autolysosomes (Li et al., 2017) and p62 expression is indicative of autophagic degradation (Fu et al., 2019). Together these proteins aid in the decomposition, recycling, and homeostasis of intracellular components (Knuppertz et al., 2014). Through the experimental results we found that NBP attenuated expression of Beclin1, CTSD, and LC3 and increased p62 expression, suggesting NBP may improve multiterritory perforator survival by inhibiting autophagy. While basal levels of autophagy aid in homeostatic maintenance, dysregulated autophagy can be pathogenic (Mao et al., 2013). One study by Wang et al. reported that excessive autophagy inhibits the pro-angiogenic effect of MTP on BM-EPC (Wang C et al., 2018). Other studies have also shown that excessive autophagy can increase the production of ROS leading to increased tissue damage and apoptosis (Maiuri et al., 2007; Scherz-Shouval and Elazar, 2011). Interestingly, in the investigation of the drug concentration gradient (Figures 1A,B), we found that as the concentration increases, skin flap necrosis does not decrease but rather further increases, which may be related to the excessive inhibitory effect of NBP on autophagy. Therefore, it is further confirmed that NBP in the perforator flap is to regulate autophagy and keep it at a low level to promote flap survival. Interestingly, in the flap research, there are several articles that have proved that the promotion of autophagy for random flaps may be beneficial to its survival (Lin et al., 2017; Chen et al., 2018; Li et al., 2019; Lin et al., 2019). The reason for the difference may be related to the difference in the construction of skin flap models. In perforator flaps, studies have shown that autophagy can inhibit the formation of microvessels in the choke Ⅱzone and also cause damage to vascular endothelial cells. Inhibition of autophagy can improve endothelial cell damage and improve angiogenesis. Therefore, autophagy has different effects in the two flap models (Jin et al., 2018).

To examine the role of autophagy in multiterritory perforator flap survival and elucidate NBP-specific autophagy effects, we used the autophagy inducer rapamycin in combination with NBP. We found that all groups treated with rapamycin, regardless of NBP treatment, exhibited features consistent with an excessive autophagic state, namely, the area of flap necrosis increased, angiogenesis decreased, and apoptosis and oxidative stress increased significantly. As expected, NBP’s beneficial effect on flap survival was abrogated when coadministered with rapamycin. These findings align with prior studies showing that inhibiting autophagy can promote the survival of multiterritory perforator flap (Wang et al., 2017; Jin et al., 2018).

Naturally, there are several limitations of the present study that still need to be further investigated. NBP is mainly used in ischemic stroke. Application in the field of skin flaps may be accompanied by side effects of other systems. However, the distal necrosis of the skin flap seriously hinders the development of reconstructive surgery, and the NBP animal experiment has shown that it can reduce the distal necrosis of the skin flap, which provides a new solution to the clinical problem. Even if it has side effects, its development and utilization value is of great significance to the solution of distal flap necrosis. In our follow-up experiments, we will further explore the effect of NBP on the systemic effects and promote its clinical transformation in the field of skin flaps.

## Conclusion

We found that NBP inhibits autophagy, improves angiogenesis, reduces oxidative stress and apoptosis, and promotes flap survival in a rat model of multiterritory perforator flap. Therefore, NBP inhibition of autophagy may be a new mechanism for improving the survival of multiterritory perforator flap.

## Materials and Methods

### Animals

One hundred and twenty male Sprague–Dawley rats (250–300 g) were purchased from the Laboratory Animal Center of Wenzhou Medical University (license no. SCXK[ZJ]2015-0001). All experimentation and care for the animals were performed under the guidance of the Laboratory Animals of the Chinese Academy of Health (wydw 2017–0022). Standard experimental cages were provided for each rat, with a 12-h light-dark cycle, enough food, and water*.*

### Reagents and Antibodies

NBP (C_₁₂_H_₁₄_O_₂_; purity, 99.98%), rapamycin, H&E staining kit, DAB developer, and pentobarbital sodium were provided by Solarbio Science & Technology (Beijing, China). The primary antibody against CDH5 was acquired from Boster Biological Technology (A02632-2; Hangzhou, China). GAPDH, SOD1, MMP9, HO1, CTSD, and CASP3 were acquired from Proteintech Group (60004-1-Ig, 10269-1-AP, 12452-1-AP, 10375-2-AP, 10701-1-AP, 21327-1-AP, and 19677-1-AP; Chicago, United States). Antibodies for p62, CYC, Bax, eNOS, and LC3 were purchased from Cell Signaling Technology (5114T 4272T, 14796, 32027, and 3868; Beverly, MA, United States). HRP-conjugated IgG secondary antibody and FITC-conjugated IgG secondary antibodies were obtained from Affinity Biosciences (s0001, s0002; Jiangsu, China). Other reagents include DAPI solution (Beyotime Biotechnology, Jiangsu, China, a BCA Kit (Thermo Fisher Scientific, Rockford, IL, United States) and an ECL Plus Reagent Kit (PerkinElmer Life Sciences, Waltham, MA, United States) which were used according to manufacturer’s instructions.

### Perforator Flap Model

**FIGURE 7:**
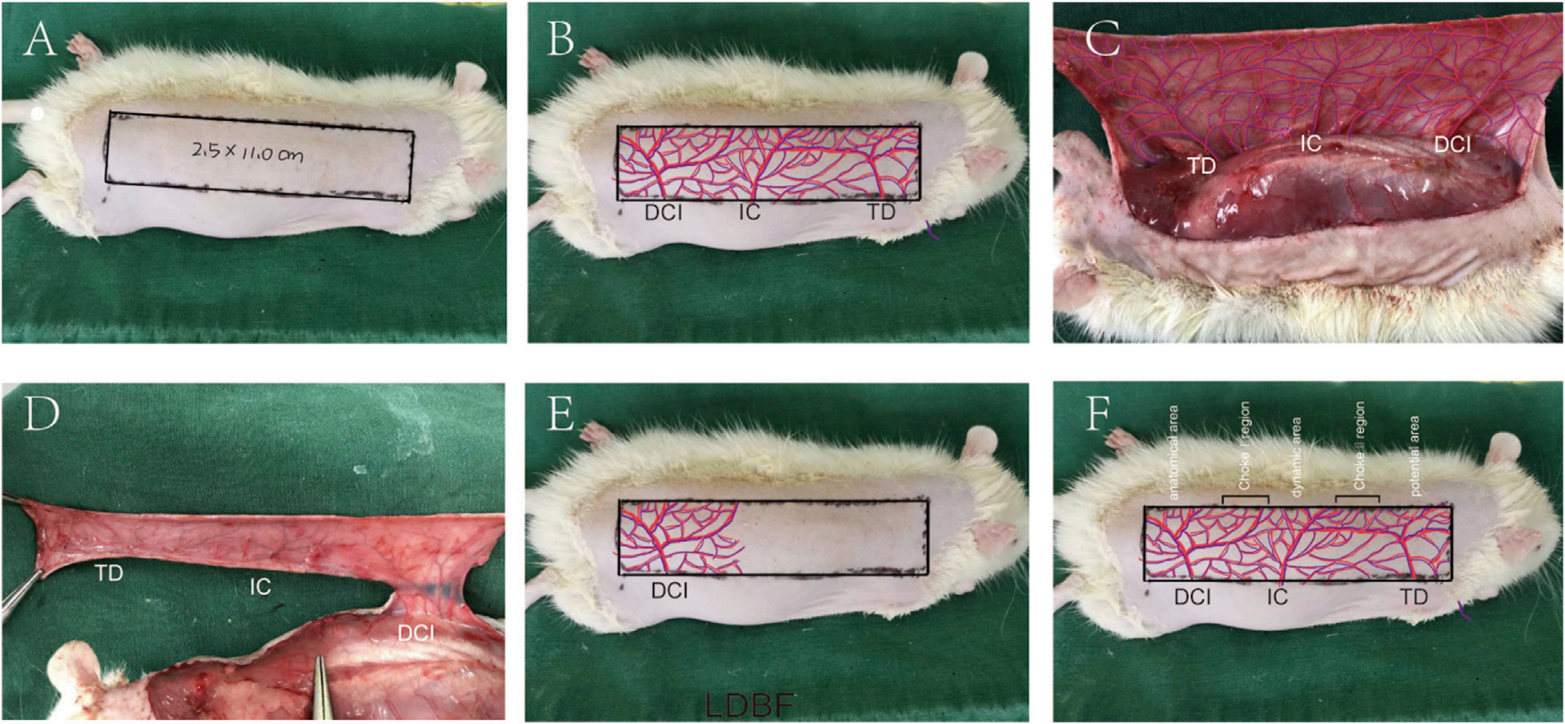
Multiterritory perforator flap procedure. **(A)** A 2.5 × 11.0 cm skin flap at the back area was marked. **(B)** Approximate distribution of involved arteries. **(C)** Exposed arteries: dorsal thoracic (TD), posterior intercostal (IC), and deep circumflex (DCI) arteries. **(D)** Excision of the TD, PIC, and preservation of the DCI. **(E)** The flap is sutured back, and its approximate blood supply range is shown. **(F)** Multiterritory perforator flap areas: anatomical, dynamic, and potential. The choke Ⅰ zone is between the anatomical and dynamic areas. The choke Ⅱ zone is between the dynamic and the potential areas.

The rats were anesthetized through intraperitoneal injection of 3% pentobarbital sodium (60 mg/kg). Then, the electric shaver and depilatory cream were used to remove the dorsal fur. Subsequent surgical steps were performed under sterile conditions. Flap size was dependent upon anatomical landmarks of rats. The medial border of the flap was based on the longitudinal axis of the spine (back midline). The lateral border was 2.5 cm away from the internal border. The caudal border was a line joining the lateral and medial border at the anterior iliac spine, forming the line of the outer boundary. The size of the flap was approximately 2.5 × 11 cm (Figure 7A). The vasculature encompassed by the flap includes the dorsal thoracic (TD), posterior intercostal (IC), and deep circumflex (DCI) arteries (Figure 7B). The flap was then separated from the fascia below. DCI blood vessels were separated and retained before TD, and IC arteries were ligated, and the flap was sutured back to its original position with 4-0 silk (Figures 7D,E). The regions where dorsal thoracic (TD), posterior intercostal (IC), and deep circumflex (DCI) arteries exist were called as potential regions, dynamic regions, and physiological regions, respectively. The multiterritory perforator flap also contains two zones, namely, choke Ⅰ zone (between physiological zone and dynamic zone) and choke Ⅱ zone (between dynamic zone and potential zone) (Figure 7F).

### Drug Administration

Ninety-six rats were randomly divided into control group (*n* = 24), NBP group (*n* = 24), NBP + Rap group (*n* = 24), and Rap group (*n* = 24). The rats in NBP and NBP + Rap group were treated with NBP at a dosage of 4.5 mg/kg/d (Zhang P et al., 2016) by intraperitoneal injection. The NBP + Rap (1.0 mg/kg/d) (Xiao et al., 2011) group received rapamycin less than 1 h prior to NBP by intraperitoneal injection. Rats in the control group received an equal volume of DMSO solution. All injections were performed for 7 days until all animals were euthanized.

### Flap Survival Assessment

Flap characteristics including color and possible necrosis were formally evaluated seven postoperative days (*n* = 6). Survival areas were evaluated using photographs of the perforator flap. ImageJ was used to quantify the survival of the flap (the range of living area/total area × 100%).

### *In Vivo* Blood Flow Imaging

Blood flow within the flap can be measured using Laser Doppler. Seven days after surgery, rats (*n* = 6) from each group were evaluated using a (Moor Instruments, Axminster, United Kingdom) laser Doppler scanner, while anesthetized. Doppler evaluation of blood supply is quantified using Moor LDI Review software (ver.6.1; Moor Instruments). We took the distal end of the DCAI vascular pedicle as the boundary and measured 6 and 8 cm, respectively, to form a rectangular area. The rectangular part is used as the measurement area. Each animal is measured three times. The measured results are used for further statistical analysis of the average blood flow value.

### Lead Oxide Angiography

On the 7th day after the operation, the rats (*n* = 6) underwent whole body lead oxide angiography. Lead oxide-gelatin (80 ml/kg) was injected into the blood vessel through the carotid artery until the limbs turn orange. Then, the perfused rat was stored at −80°C overnight. On the second day, the rats were thawed and the flaps were collected, and X-ray machine (54 kVp, 40 mA, 100 s) was used for vascular imaging. The blood vessel condition of the choke Ⅱ zone reflects the effect of drug intervention (Supplementary Figure S2).

### Microscopy

Rats were euthanized at seven days after surgery. Then, flap samples (*n* = 6) of the choke Ⅱ zone (we took the distal end of the DCAI vascular pedicle as the boundary and measured 6 and 8 cm, respectively, to form a rectangular area; the middle part of the rectangular area serves as the tissue collection site) in each group were collected. Samples were fixed in 4% paraformaldehyde for one day, prior to paraffin embedding in 4 mm thick slices of paraffin wax. Sections were fixed on poly-L-lysine-coated slides for H&E. Vessel density was measured using an optical microscope (Olympus Corp, Tokyo, Japan) by counting the number of microvessels per mm^2^ tissue.

Six samples from each group were randomly selected from the paraffin sections, dewaxed using xylene, and rehydrated using a graded ethanol series. Sections were then washed and blocked using 3% (v/v) H_2_O_2_ and kept at 95°C for 20 min. Antigen recovery was performed using a 10.2 mM sodium citrate buffer (pH 6.0). Sections were blocked with 10% (w/v) bovine serum albumin phosphate buffer for 10 min before incubation at 4°C overnight with a primary antibody targeting CD34 (1: 100), SOD1 (1: 100), CTSD (1:100), VEGF (1: 200), CDH5 (1: 200), or CASP3 (1: 200). Slides were then incubated with an HRP-conjugated secondary antibody (1:1000) and counterstained with hematoxylin.

Imaging of flap tissue was conducted using a DP2-TWAN image acquisition system (Olympus, Corp, Tokyo, Japan). ImageJ was used to measure the overall absorbance of VEGF, CDH5, CASP3, SOD1, and CTSD and CD34 positive blood vessels.

### Immunofluorescence

Samples of the choke Ⅱ zone in each group were selected and treated for antigen retrieval as above, prior to permeabilization using 0.1% (v/v) PBS-Triton X-100 (10 min) and blocking with 10% (v/v) bovine serum albumin in PBS for 1 h. Slides were then incubated at 4°C overnight with anti-LC3 (1:200) prior to evaluation using a fluorescence microscope (Olympus). The percentage of LC3 positive cells in the dermal layer was determined by counting six random fields on three random sections from each tissue sample.

### Immunoblotting

Samples (0.5 × 0.5 cm) from the choke Ⅱ zone (*n* = 6) were harvested for western blot analyses. Flap tissue proteins were extracted and quantified using a BCA assay. 60 µg of proteins was subject to electrophoresis and transferred using polyvinylidene difluoride membranes (Roche Applied Science, Indianapolis, IN, United States). This was followed by blocking using 5% (w/v) nonfat milk for 2 h at room temperature and incubation with the following primary antibodies at 4°C overnight: VEGF (1:1,000), MMP-9 (1:1,000), CDH5 (1:1,000), HO1 (1:1,000), eNOS (1:1,000), SOD1 (1:1,000), Bax (1:1000), CYC (1:1,000), Caspase 3 (CAPS3) (1:1,000), Beclin1 (1:1,000), p62 (1:1,000), LC3 (1:500), CTSD (1:1,000), and GAPDH (1:1,000). HRP-conjugated IgG secondary antibody (1:5,000) was then incubated with the membranes for 2 h at room temperature prior to evaluation using ECL Plus Reagent Kit. Band intensity was quantified using Image Lab 3.0 software (Bio-Rad, Hercules, CA, United States).

### Statistical Analyses

Quantitative experimental data are presented as mean ± standard error. Independent-sample *t*-test and one-way ANOVA with LSD (equal variances assumed) or Dunnett’s T3 (equal variances not assumed) post hoc analyses were used as appropriate. *p* < 0.05 was considered statistically significant.

## Data Availability Statement

The original contributions presented in the study are included in the article/Supplementary Material; further inquiries can be directed to the corresponding author.

## Ethics Statement

The animal study was reviewed and approved by the Animal Research Committee of Wenzhou Medical University (SCXK[ZJ]2015-0001).

## Author Contributions

BL and ZC wrote the manuscript text. BL, ZC, XL, CZ, and HC collected samples. BL, ZC, MZ, HM and JL prepared figures. SW, YY, and MC analyzed data. HY designed the experiment and revised the manuscript. All authors contributed to the article and approved the submitted version.

## Funding

This work was supported by the Zhejiang Provincial Natural Science Foundation of China (LY18H060010), Wenzhou Science and Technology Project of Zhejiang China (Y20180667), and Zhejiang Traditional Chinese Medicine Administration (2018ZB079).

## Conflict of Interest

The authors declare that the research was conducted in the absence of any commercial or financial relationships that could be construed as a potential conflict of interest.

## References

[B1] Arise R. O. Tella A. C. Akintola A. A. Akiode S. O. Malomo S. O. (2012). Toxicity evaluation of crankcase oil in rats. Excli J. 11, 219–225. 10.17877/DE290R-5759 27366138 PMC4928016

[B2] Cai W. Li Y. Yi Q. Xie F. Du B. Feng L. (2015). Total saponins from Albizia julibrissin inhibit vascular endothelial growth factor-mediated angiogenesis *in vitro* and *in vivo* . Mol. Med. Rep. 11 (5), 3405–3413. 10.3892/mmr.2015.3228 25607254 PMC4368075

[B3] Chen G. Shen H. Zang L. Su Z. Huang J. Sun Y. (2018). Protective effect of luteolin on skin ischemia-reperfusion injury through an AKT-dependent mechanism. Int. J. Mol. Med. 42 (6), 3073–3082. 10.3892/ijmm.2018.3915 30280183 PMC6202092

[B4] Chen L. Zhou K. Chen H. Li S. Lin D. Zhou D. (2017). Calcitriol promotes survival of experimental random pattern flap via activation of autophagy. Am. J. Transl. Res. 9 (8), 3642–3653. 28861155 PMC5575178

[B5] Chen X. Wang L. Deng Y. Li X. Li G. Zhou J. (2019). Inhibition of autophagy prolongs recipient survival through promoting CD8. Front. Immunol. 10, 1356 10.3389/fimmu.2019.01356 31258533 PMC6587890

[B6] Fichter A. M. Ritschl L. M. Robitzky L. K. Wagenpfeil S. Mitchell D. A. Wolff K.-D. (2016). Impact of different antithrombotics on the microcirculation and viability of perforator-based ischaemic skin flaps in a small animal model. Sci. Rep. 6 35833 10.1038/srep35833 27767060 PMC5073281

[B7] Fimia G. M. Stoykova A. Romagnoli A. Giunta L. Di Bartolomeo S. Nardacci R. (2007). Ambra1 regulates autophagy and development of the nervous system. Nature. 447 (7148), 1121–1125. 10.1038/nature05925 17589504

[B8] Fu C. Liu P. Li P. Liu W. Huang X. Liang Y. (2019). FSP1 promotes the biofunctions of adventitial fibroblast through the crosstalk among RAGE, JAK2/STAT3 and Wnt3a/β-catenin signalling pathways. J. Cell Mol. Med. 23 (11), 7246–7260. 10.1111/jcmm.14518 31454154 PMC6815850

[B9] Fujioka M. (2014). Surgical reconstruction of radiation injuries. Adv. Wound Care. 3 (1), 25–37. 10.1089/wound.2012.0405 PMC3900101 24761342

[B10] Guo K. Ma J. Liang W. (2018). Effects of SB202190 on expression levels of IL-6 and NF-κB in flap ischemia-reperfusion injury. Exp. Ther. Med. 16 (3), 2522–2526. 10.3892/etm.2018.6442 30210603 PMC6122530

[B11] Gupta A. Puri S. Puri V. (2019). Bioinformatics unmasks the maneuverers of pain pathways in acute kidney injury. Sci. Rep. 9 (1), 11872 10.1038/s41598-019-48209-x 31417109 PMC6695489

[B12] Hou P. Yang K. Jia P. Liu L. Lin Y. Li Z. (2020). A novel selective autophagy receptor, CCDC50, delivers K63 polyubiquitination-activated RIG-I/MDA5 for degradation during viral infection. Cell Res. 10.1038/s41422-020-0362-1 PMC7852694 32612200

[B13] Jin Z. Chen S. Wu H. Wang J. Wang L. Gao W. (2018). Inhibition of autophagy after perforator flap surgery increases flap survival and angiogenesis. J. Surg. Res. 231, 83–93. 10.1016/j.jss.2018.05.018 30278973

[B14] Knuppertz L. Hamann A. Pampaloni F. Stelzer E. Osiewacz H. D. (2014). Identification of autophagy as a longevity-assurance mechanism in the aging model Podospora anserina. Autophagy. 10 (5), 822–834. 10.4161/auto.28148 24584154 PMC5119060

[B15] Li J. Bao G. E A. L. Ding J. Li S. Sheng S. (2019). Betulinic acid enhances the viability of random-pattern skin flaps by activating autophagy. Front. Pharmacol. 10, 1017 10.3389/fphar.2019.01017 31572190 PMC6753397

[B16] Li Y. Chang Y. Ye N. Dai D. Chen Y. Zhang N. (2017). Advanced glycation end products inhibit the proliferation of human umbilical vein endothelial cells by inhibiting cathepsin D. Int. J. Mol. Sci. 18 (2), 436 10.3390/ijms18020436 PMC5343970 28218663

[B17] Li Y. Zhao X. Hu Y. Sun H. He Z. Yuan J. (2018). Age-associated decline in Nrf2 signaling and associated mtDNA damage may be involved in the degeneration of the auditory cortex: implications for central presbycusis. Int. J. Mol. Med. 42 (6), 3371–3385. 10.3892/ijmm.2018.3907 30272261 PMC6202109

[B18] Lin D. Wu H. Zhou Z. Tao Z. Gao W. Jia T. (2020). The effect of leonurine on multiterritory perforator flap survival in rats. J. Surg. Res. 245, 453–460. 10.1016/j.jss.2019.07.085 31445497

[B19] Lin J. Lin R. Li S. Wu H. Ding J. Xiang G. (2019). Protective effects of resveratrol on random-pattern skin flap survival: an experimental study. Am. J. Transl. Res. 11 (1), 379–392. 30787995 PMC6357324

[B20] Lin J. Lin R. Li S. Wu H. Ding J. Xiang G. (2018). Salvianolic acid B promotes the survival of random-pattern skin flaps in rats by inducing autophagy. Front. Pharmacol. 9, 1178 10.3389/fphar.2018.01178 30405410 PMC6206168

[B21] Lin R. Chen H. Callow D. Li S. Wang L. Li S. (2017). Multifaceted effects of astragaloside IV on promotion of random pattern skin flap survival in rats. Am. J. Transl. Res. 9, 4161–4172. 28979690 PMC5622259

[B22] Liu R. Z. Fan C. X. Zhang Z. L. Zhao X. Sun Y. Liu H. H. (2017). Effects of dl-3-n-butylphthalide on cerebral ischemia infarction in rat model by mass spectrometry imaging. Int. J. Mol. Sci. 18 (11), 2451 10.3390/ijms18112451 PMC5713418 29165327

[B23] Luo X. Liu J. Chen H. Li B. Jin Z. Zhao M. (2020). The feasibility and survival mechanism of a large free flap supported by a novel hybrid perfusion mode. Oral Oncol. 101, 4506 10.1016/j.oraloncology.2019.104506 31863964

[B24] Maiuri M. C. Zalckvar E. Kimchi A. Kroemer G. (2007). Self-eating and self-killing: crosstalk between autophagy and apoptosis. Nat. Rev. Mol. Cell Biol. 8 (9), 741–752. 10.1038/nrm2239 17717517

[B25] Mao K. Chew L. H. Inoue-Aono Y. Cheong H. Nair U. Popelka H. (2013). Atg29 phosphorylation regulates coordination of the Atg17-Atg31-Atg29 complex with the Atg11 scaffold during autophagy initiation. Proc. Natl. Acad. Sci. U. S. A. 110 (31), E2875–E2884. 10.1073/pnas.1300064110 23858448 PMC3732952

[B26] Mărginean C. O. Mărginean C. Bănescu C. Meliţ L. E. Tripon F. Iancu M. (2019). The relationship between MMP9 and ADRA2A gene polymorphisms and mothers-newborns’ nutritional status: an exploratory path model (STROBE compliant article). Pediatr. Res. 85 (6), 822–829. 10.1038/s41390-019-0347-2 30791043 PMC6760549

[B27] Marunouchi T. Tanonaka K. (2015). Cell death in the cardiac myocyte. Biol. Pharm. Bull. 38 (8), 1094–1097. 10.1248/bpb.b15-00288 26235571

[B28] Mizushima N. Yoshimori T. Ohsumi Y. (2011). The role of Atg proteins in autophagosome formation. Annu. Rev. Cell Dev. Biol. 27, 107–132. 10.1146/annurev-cellbio-092910-154005 21801009

[B29] Scherz-Shouval R. Elazar Z. (2011). Regulation of autophagy by ROS: physiology and pathology. Trends Biochem. Sci. 36 (1), 30–38. 10.1016/j.tibs.2010.07.007 20728362

[B30] Shi J. Gu J. H. Dai C. L. Gu J. Jin X. Sun J. (2015). O-GlcNAcylation regulates ischemia-induced neuronal apoptosis through AKT signaling. Sci. Rep. 5, 14500 10.1038/srep14500 26412745 PMC4585968

[B31] Song F. X. Wang L. Liu H. Wang Y. L. Zou Y. (2017). Brain cell apoptosis inhibition by butylphthalide in Alzheimer's disease model in rats. Exp. Ther. Med. 13 (6), 2771–2774. 10.3892/etm.2017.4322 28587340 PMC5450572

[B32] Wang C. Mao C. Lou Y. Xu J. Wang Q. Zhang Z. (2018). Monotropein promotes angiogenesis and inhibits oxidative stress-induced autophagy in endothelial progenitor cells to accelerate wound healing. J. Cell Mol. Med. 22 (3), 1583–1600. 10.1111/jcmm.13434 29278309 PMC5824424

[B33] Wang L. Jin Z. Wang J. Chen S. Dai L. Lin D. (2017). Detrimental effect of Hypoxia-inducible factor-1α-induced autophagy on multiterritory perforator flap survival in rats. Sci. Rep. 7 (1), 11791 10.1038/s41598-017-12034-x 28924179 PMC5603514

[B34] Wang Z. Cui R. Wang K. (2018). Effects of sevoflurane pretreatment on the apoptosis of rat H9c2 cardiomyocytes and the expression of GRP78. Exp. Ther. Med. 15 (3), 2818–2823. 10.3892/etm.2018.5799 29599827 PMC5867468

[B35] Xiao B. Xia W. Zhao K. Yang Y. Fu S. Li Y. (2011). *Ex vivo* transfer of adenovirus-mediated CTLA4Ig gene combined with a short course of rapamycin therapy prolongs free flap allograft survival. Plast. Reconstr. Surg. 127 (5), 1820–1829. 10.1097/PRS.0b013e31820cf264 21532411

[B36] Xiong Z. Lu W. Zhu L. Zeng L. Shi C. Jing Z. (2017). Dl-3-n-Butylphthalide treatment enhances hemodynamics and ameliorates memory deficits in rats with chronic cerebral hypoperfusion. Front. Aging Neurosci. 9, 238 10.3389/fnagi.2017.00238 28798681 PMC5526838

[B37] Yang N. Wang L. Liu J. Liu L. Huang J. Chen X. (2018). MicroRNA-206 regulates the epithelial-mesenchymal transition and inhibits the invasion and metastasis of prostate cancer cells by targeting Annexin A2. Oncol. Lett. 15 (6), 8295–8302. 10.3892/ol.2018.8395 29805562 PMC5950137

[B38] Ye Z. Y. Xing H. Y. Wang B. Liu M. Lv P. Y. (2019). DL-3-n-butylphthalide protects the blood-brain barrier against ischemia/hypoxia injury via upregulation of tight junction proteins. Chin. Med. J. 132 (11), 1344–1353. 10.1097/cm9.0000000000000232 30939485 PMC6629356

[B39] Yu Y. Duan J. Yu Y. Li Y. Liu X. Zhou X. (2014). Silica nanoparticles induce autophagy and autophagic cell death in HepG2 cells triggered by reactive oxygen species. J. Hazard Mater. 270, 176–186. 10.1016/j.jhazmat.2014.01.028 24583672

[B40] Zhang P. Guo Z. F. Xu Y. M. Li Y. S. Song J. G. (2016). N-Butylphthalide (NBP) ameliorated cerebral ischemia reperfusion-induced brain injury via HGF-regulated TLR4/NF-κB signaling pathway. Biomed. Pharmacother. 83, 658–666. 10.1016/j.biopha.2016.07.040 27468961

[B41] Zhang Y. Cai X. Shen L. Huang X. Wang X. Lan Y. (2016). Effects of sanguis draconis on perforator flap survival in rats. Molecules. 21 (10), 1262 10.3390/molecules21101262 PMC6273294 27681718

[B42] Zhao F. Zhang D. Zhao Y. Wang W. Yang H. Tai F. (2016). The difference of physiological and proteomic changes in maize leaves adaptation to drought, heat, and combined both stresses. Front. Plant Sci. 7, 1471 10.3389/fpls.2016.01471 27833614 PMC5080359

[B43] Zhong R. Chen Q. Zhang X. Li M. Lin W. (2019). L-3-n-butylphthalide soft capsules in the treatment of Parkinson disease dementia: a systematic review and meta-analysis of randomized controlled trials. Medicine. 98 (24), e16082 10.1097/md.0000000000016082 31192971 PMC6587622

[B44] Zhou F. Zhang L. Chen L. Xu Y. Chen Y. Li Z. (2019). Prevascularized mesenchymal stem cell-sheets increase survival of random skin flaps in a nude mouse model. Am. J. Transl. Res. 11 (3), 1403–1416. 30972170 PMC6456548

[B45] Zhu X. Chen Y. Chen Q. Yang H. Xie X. (2018). Astaxanthin promotes Nrf2/ARE signaling to alleviate renal fibronectin and collagen IV accumulation in diabetic rats. J Diabetes Res. 2018, 6730315 10.1155/2018/6730315 29744366 PMC5884145

