# Supplementary figures and images for: Butylphthalide Inhibits Autophagy and Promotes Multiterritory Perforator Flap Survival
Source: Front Pharmacol. 2021 Jan 29;11:612932. doi: 10.3389/fphar.2020.612932 (PMC7878674; doi:10.3389/fphar.2020.612932)

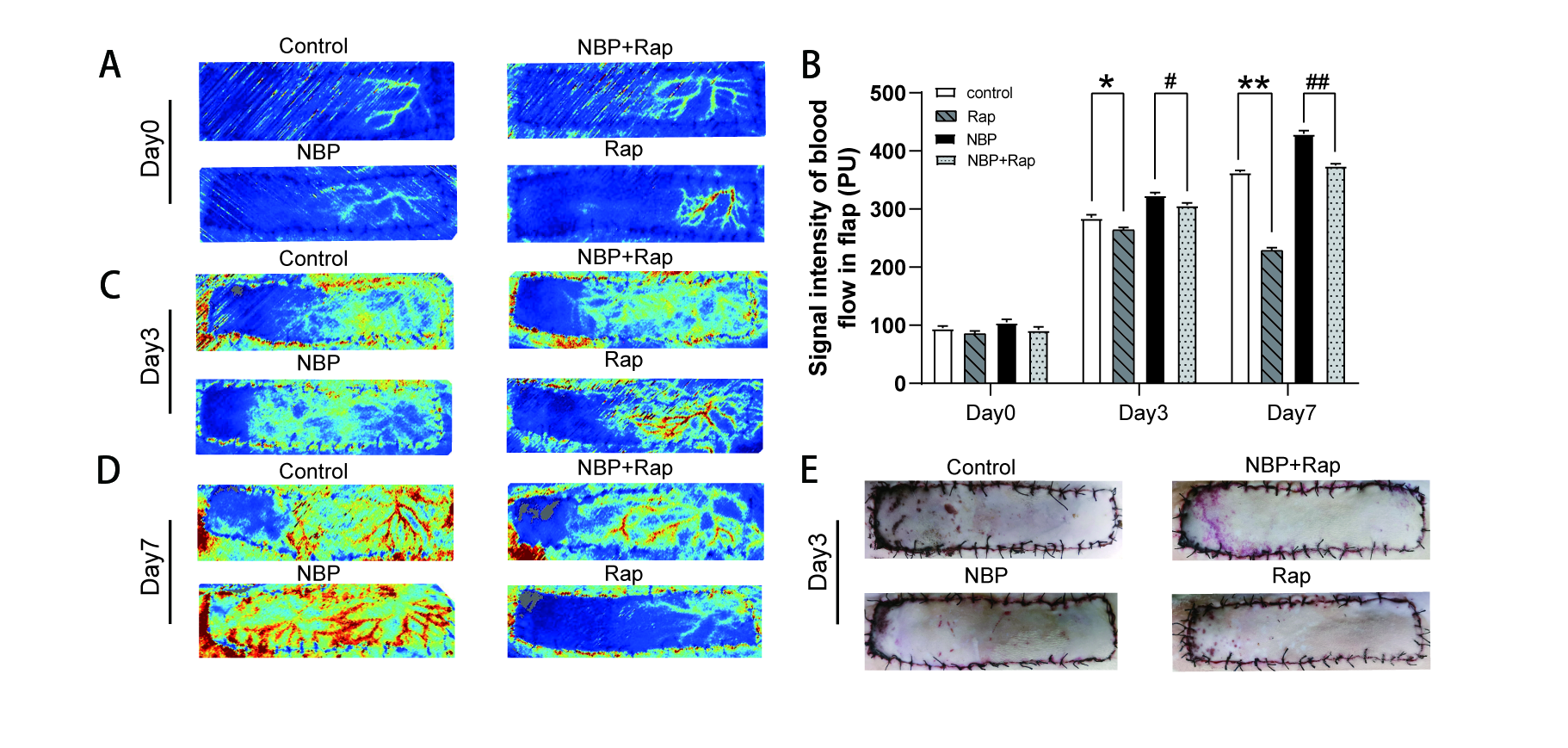

Supplement: Supplementary file 1 [file image1.tif]

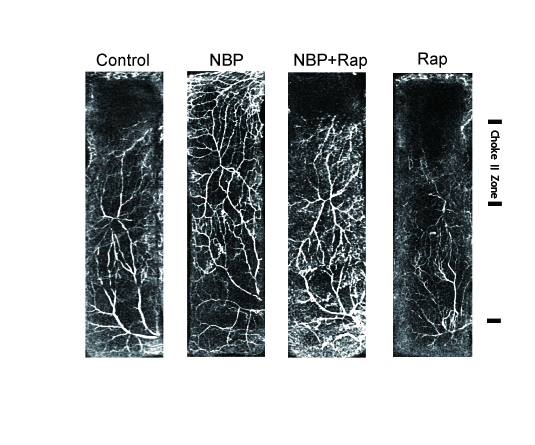

Supplement: Supplementary file 2 [file image2.tif]
